# Phenolic Compounds and Capsaicinoids in Three *Capsicum annuum* Varieties: From Analytical Characterization to In Silico Hypotheses on Biological Activity

**DOI:** 10.3390/molecules28196772

**Published:** 2023-09-22

**Authors:** Deborah Giordano, Angelo Facchiano, Paola Minasi, Nunzio D’Agostino, Mario Parisi, Virginia Carbone

**Affiliations:** 1 Institute of Food Sciences, National Research Council, Via Roma 64, 83100 Avellino, Italy; deborah.giordano@isa.cnr.it (D.G.); paola.minasi@isa.cnr.it (P.M.); 2 Department of Agricultural Sciences, University of Naples Federico II, 80055 Portici, Italy; nunzio.dagostino@unina.it; 3 CREA Research Centre for Vegetable and Ornamental Crops, Via Cavalleggeri 25, 84098 Pontecagnano, Italy; mario.parisi@crea.gov.it

**Keywords:** *Capsicum annuum*, phenolic compounds, capsaicinoids, HPLC-MS, transient receptor potential vanilloid member 1 (TRPV1), TRPV1–capsaicin interaction, protein modelling, docking simulations

## Abstract

The affinity of specific phenolic compounds (PCs) and capsaicinoids (CAPs) present in three *Capsicum annuum* varieties (Friariello, Cayenne and Dzuljunska Sipka) to the transient receptor potential vanilloid member 1 (TRPV1) was investigated by integrating an analytic approach for the simultaneous extraction and analysis through high-performance liquid chromatography coupled with ion trap mass spectrometry (HPLC/ITMS) and UV detection (HPLC-UV) of PCs and CAPs and structural bioinformatics based on the protein modelling and molecular simulations of protein–ligand docking. Overall, a total of 35 compounds were identified in the different samples and CAPs were quantified. The highest content of total polyphenols was recorded in the pungent Dzuljunska Sipka variety (8.91 ± 0.05 gGAE/Kg DW) while the lowest was found in the non-pungent variety Friariello (3.58 ± 0.02 gGAE/Kg DW). Protein modelling generated for the first time a complete model of the homotetrameric human TRPV1, and it was used for docking simulations with the compounds detected via the analytic approach, as well as with other compounds, as an inhibitor reference. The simulations indicate that different capsaicinoids can interact with the receptor, providing details on the molecular interaction, with similar predicted binding energy values. These results offer new insights into the interaction of capsaicinoids with TRPV1 and their possible actions.

## 1. Introduction

The *Capsicum* genus (Solanaceae family) is native to South America and includes over 30 species, five of which have been domesticated: *C. annuum*, *C. baccatum*, *C. chinense*, *C. frutescens* and *C. pubescens* [1]. Peppers are grown all over the world, and their annual production has increased significantly over the years. The cultivated area covers 3.7 million hectares with a total production of about 41 million tons (FAOSTAT 2021) [2]. A tremendous wealth of genetic variation in fruit size and shape [3], colour [4] and flavour [5,6,7,8] is known within the *Capsicum* species. Today, pepper fruits are used for different purposes as fresh, dehydrated or processed vegetables and spices in medicine, pest and animal control and even in law enforcement, giving this crop immense cultural and economic importance [9]. Peppers are good sources of ascorbic acid (vitamin C) and other phytochemicals, such as polyphenols, including flavonoids and carotenoids [10]. Phenolic compounds are abundant secondary metabolites in plants. Their great chemical variability includes several thousands of compounds from simple phenolic acids to complex flavonoids [11]. Different lines of evidence indicate that the use of a diet rich in phenolic compounds reduces the risk of chronic diseases. Due to their ability to inactivate or prevent the formation of reactive free radicals, polyphenolic compounds have antioxidant properties, although some of them are also regarded as antimicrobial or anticarcinogenic [11,12]. The carotenoid and anthocyanin pigments are responsible for the fruit colour and for the nutritional value of the pepper fruits. The colour of each Capsicum variety is variable, starting from green, yellow or white in the case of the unripe fruit and turning to red, dark red, brown, and sometimes almost black at the fully ripe stage [13]. The predominant red pigments are capsanthin and capsorubin, and the yellow and orange pigments are lutein, β-carotene (provitamin A), zeaxanthin, violaxanthin and antheraxanthin [14]. All these compounds found in Capsicum provide many nutritional and health benefits that include antioxidant, anti-inflammatory, and antimicrobial activities, reduced prevalence of type 2 diabetes and obesity, protection against hypercholesterolemia, reduced prevalence of atherosclerotic cardiovascular diseases and a protective effect on some pro-oxidants that can induce lipid peroxidation in brain and liver [15,16,17,18]. The amount of these compounds in peppers depends on many factors, including cultivar, maturity, growing conditions and climate [10,19,20].

An important distinctive feature of pepper fruits is the presence or absence of pungency. Hot peppers are called chiles, chillis or chilis; conversely, non-pungent varieties are referred to as sweet peppers, although the sugar content can vary greatly in the fruit. Hot peppers are characterised by different levels of pungency and other aroma and flavour molecules [21]. Hot peppers are used fresh or dried in various pharmacological preparations and are widely used in cooking to enrich foods with their unique flavour. The spiciness of chili peppers is due to the presence of lipophilic alkaloids at different concentrations, collectively called capsaicinoids [22]. Capsaicin and dehydrocapsaicin are the predominant molecules, representing ~90% of the total capsaicinoid content, and usually, the evaluation of their quantity is fundamental for determining pungency. Some additional related compounds, such nordihydrocapsaicin, homocapsaicin and homodihydrocapsaicin, are also present in minor concentrations in the fruits [23]. Capsaicinoids are synthesized and accumulated in the epidermal cells of the placenta and are transported into the apoplast and stored in the vesicles of the placenta, also called “blisters” [24]. Several health benefits have been associated with polyphenols and capsaicinoids present in different *Capsicum* genotypes and include antioxidant, antimicrobial, anti-inflammatory, antihypertensive, antihyperglycemic, metal chelating and antitumoral activities [25].

The affinity of capsaicin with the transient receptor potential vanilloid member 1 (TRPV1) has been the subject of several studies. TRPV1 is a homotetrameric calcium-permeable ion channel expressed mainly in neuronal cells, such as trigeminal nerves, dorsal region ganglia, central nerves, and peripheral sensory nerve endings [26]. TRPV1 is known to also be present in non-neural tissues, including vascular smooth muscle cells [27], lung tissues, and cells of the immune system where it plays a key role in inflammation and immunity [28]. Different agents can activate TRPV1: high temperature; extracellular osmolality alterations; extracellular acidification; vanilloid compounds, such as capsaicin or dihydrocapsaicin; or other compounds, such as arachidonic derivative or piperine. Additionally, many natural (oleic acid) and synthetic (SB-366791) compounds with an antagonist role are described [26].

Each TRPV1 monomer is composed of a repeating ankyrin domain in the N-terminal region, a transmembrane domain in the core region, with six transmembrane helices per subunit (S1 to S6) forming the pore of the channel, and a C-terminal domain; both N- and C- terminal regions are located intracellularly [29]. When TRPV1 is activated, calcium or sodium ions flow into the cell depolarizing the nociceptive neurons, resulting in the spicy sensation; however, TPRV1 activation is involved in the modulation of certain processes, such as synaptic transmission, temperature regulation, pain perception and apoptosis [30].

In this work, an analytic approach for the simultaneous extraction and analysis via high-performance liquid chromatography coupled with ion trap mass spectrometry (HPLC/ITMS) and UV detection (HPLC-UV) of phenolic compounds (PCs) and capsaicinoids (CAPs) in three varieties of *Capsicum annuum* was integrated withstructural bioinformatics, based on protein modelling and molecular simulations of protein–ligand docking, to verify the affinity of compounds to TRPV1. The results suggest possible molecular mechanisms underlying TRPV1 activation or inhibition, with more capsaicinoids able to bind the TRPV1 receptor with comparable values of predicted interaction energy.

## 2. Results and Discussion

### 2.1. Qualitative and Quantitative Analysis of Phenolic Compounds and Capsaicinoids in Pepper Fruit Extracts

The highest content of total polyphenols was recorded in RP3 (8.91 ± 0.05 g_GAE_/Kg DW); the lowest was found in GP (3.58 ± 0.02 g_GAE_/Kg DW) (Table 1).

**Figure 1:**
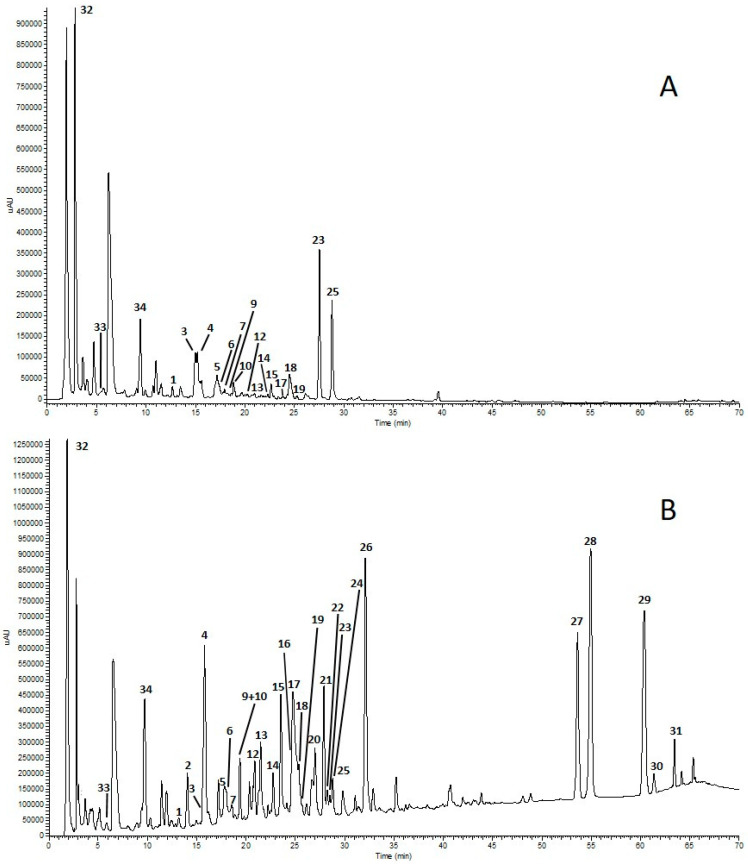
HPLC-UV chromatograms of extracts from green pepper (GP) (**A**) and red pepper 3 (RP3) (**B**) recorded at 280 nm. For peak assignments, see Table 2.

HPLC-UV and HPLC-ESI-ITMS analysis allowed the identification of phenolic compounds and capsaicinoids in pepper fruit extracts. HPLC-UV chromatograms of GP and RP3 samples are shown in Figure 1A,B.

Overall, a total of 35 compounds were identified in the different samples on the basis of their pseudomolecular [M-H]^−^ ions, together with the interpretation of their collision-induced dissociation (CID) fragments. When authentic standards were available, identification was conducted by comparing retention times and fragmentation spectra with those of the standards.

The classes of compounds detected agreed with those already reported in previous studies on Capsicum species [25,31,32,33,34,35,36] and included hydroxycinnamic acids, lignans, flavones and flavonols in all the four samples analysed as well as capsaicinoids in the three pungent *C. annuum* samples (Table 2).

Furthermore, in all analysed samples, we found the lignan glycoside icariside E5, isolated for the first time in 1989 in a plant of the genus Epimedium (*E. diphyllum*) [37] and found by Iorizzi et al. (2001) [38] in ripe fruits of *Capsicum annuum* L. var. *acuminatum*. In particular, Iorizzi et al. (2001) demonstrated that icariside E5, while not showing capsaicin-like activity, significantly prevented serum withdrawal-induced apoptosis in Jurkat cells, indicating a potential antioxidant role in cultured cells.

The HPLC/ESI-ITMS^n^ analyses of RP3 extract showed a compound with pseudomolecular ion [M-H]^−^ at *m*/*z* 593 in peak 20 (Figure 1B; Table 2) that was tentatively identified as luteolin deoxyhexosylhexoside, according to MS fragmentation, leading to the luteolin aglycone at *m*/*z* 285 after a sequential loss of deoxyhexose and hexose moieties [M-H^−^ 146–162]. Interestingly, apigenin 7-*O*-(malonylapiosyl) hexoside (Peak 26; Figure 1B; Table 2) was previously found in extracts of the leaves of *C. chinense* [39] and in the leaves of several *Capsicum* species [40], but, to the best of our knowledge, the presence of this phenolic compound in the extracts of pungent pepper fruits is reported here for the first time. As for the quantitative analyses of capsaicinoids, capsaicin is the most abundant compound in all three hot pepper samples (RP1, RP2 and RP3), followed by dihydrocapsaicin and nordhydrocapsaicin. Furthermore, RP3 has the highest amount of total capsaicinoids (40.75 ± 0.54 g/kg of DW), as shown in Table 3.

### 2.2. Human TRPV1 Model

**Figure 2:**
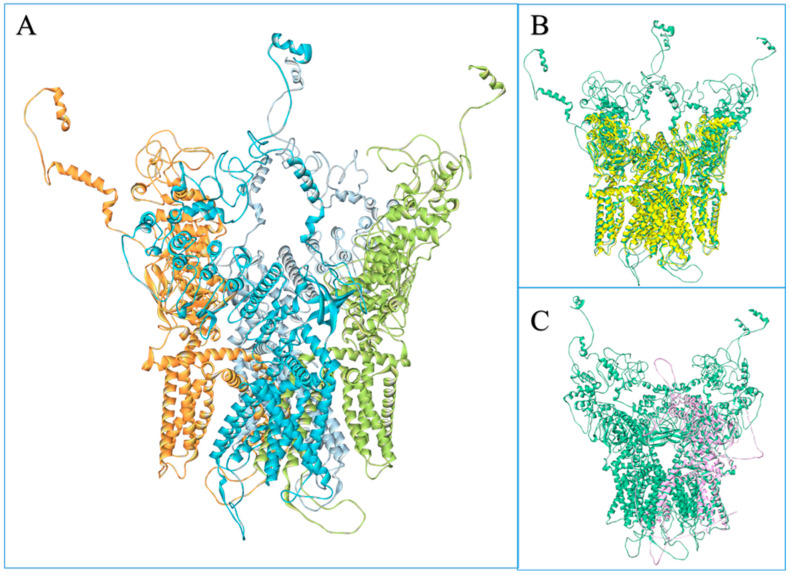
Model of the human TRPV1. (**A**) The ‘cartoon’ representation of the homotetrameric model of TPRV1, coloured by chain. (**B**) Overlap between the model of TRPV1 (green) and the template (PDB code: 7LPB) (cartoon). (**C**) Overlap between the chain A of the TRPV1 model (green) and the single chain of the Alpha Fold model (pink).

The model of the complete structure of the homo-tetrameric human TRPV1 receptor in its agonist-bound conformation was obtained for the first time in this study (Figure 2). The crystal structures available in the Protein Data Bank (PDB) lack the C and N terminal regions and often have some regions missing in the core region as well; moreover, the available AlphFold model is monomeric and unfolded at the ends. Comparing the quality of the model constructed with the template and the AlphaFold monomer, it is found that the template has a Z-score equal to −5.9 for each of its chains and the Ramachandran plot values of 87.8% core; 12% allowed; 0.2% generously allowed; and 0% disallowed.

The AlphaFold single-chain model has a Z-score of −8.57, and Ramachandran plot values of 83.2%; −14.2%; −2.1%; and −0.4% (core, allowed, genereously allowed, disallowed regions, respectively). The model obtained, instead, has Z-score of −7.95 for chain A, −7.98 for chain B, −8.02 for chain C and −8.01 for chain D, with the Ramachandran plot highlighting 88.8% of dihedral angles in the most favoured regions (core), 9.2% in those allowed, 0.8% in those generously allowed and 0.6% in those disallowed. The Qmean 4 values are quite comparable: 0.650 for the template, 0.615 for the AlphaFold model and 0.678 for the TRPV1 model.

The development of automated procedures for predicting the 3D structure of proteins, and in particular, the success of AI-based methods has greatly improved the quality of the structures that can be predicted. However, great care still needs to be taken in assessing the quality of the models obtained, and in fact, the models produced using AlphaFold are accompanied by several tools to assess their quality. In this work, it was necessary to generate models for human TRPV1 with a procedure that integrated both automatic server-based predictions and modelling guided by the careful definition of alignments between templates and target sequence. The quality of the obtained model is a prerequisite for applying reliable molecular docking procedures. Moreover, it should be noted that the AlphaFold models accessible by the UniProt database are in a monomeric, apo, inactive conformation that often is not suitable to perform docking simulations, so that refinement procedures may improve these valid models on the base of suitable selected crystallographic templates [41].

### 2.3. Docking Results

Docking analyses were performed with all detected capsaicinoids, in order to investigate their ability to interact with the receptor, and with two phenolic compounds, namely the caffeic acid hexoside and the icariside E5, to test the absence of capsaicin-like activities. As positive and negative controls, capsaicin and the inhibitor SB-366791 were also tested [26]. Table 4 lists the docking results.

Since the TRPV1 agonist and antagonist share the same binding pocket, the former inducing the conformational change that leads to the pore opening and the latter inhibiting it [42], the same docking simulations were performed, exploiting a dual state of TRPV1: one bound to the inhibitor (referred to as antagonist-bound conformation in Table 4) and the other modelled to bind capsaicin (referred to as agonist-bound conformation in Table 4).

Capsaicin and SB-366791 exhibit similar binding energy for both TRPV1 conformations: −7.33 Kcal/mol and −7.25 Kcal/mol, respectively, for the agonist-bound conformation, with comparable estimated inhibition constants (Ki), and in the range of −8.20 Kcal/mol to −7.77 Kcal/mol for capsaicin and −8.12 Kcal/mol for the inhibitor binding to the antagonist-bound conformation.

**Figure 3:**
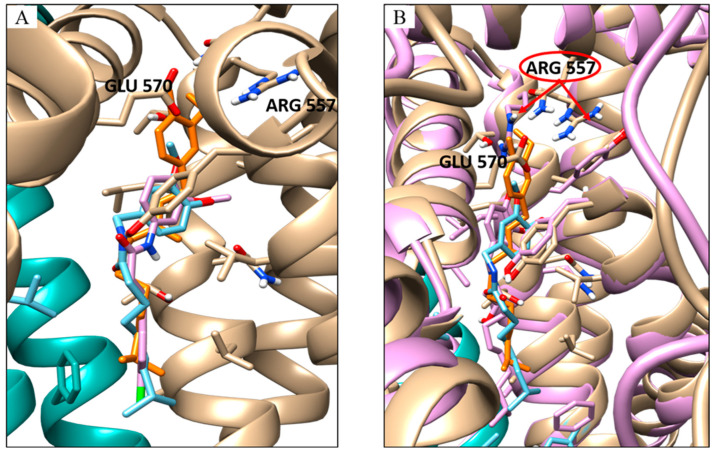
Docking results for capsaicin and inhibitor. (**A**) Overlay of the docking pose of the capsaicin with the best energy cluster (orange stick) with the capsaicin with the best numerousness in cluster (cyan stick) and the inhibitor (pink stick). (**B**) Overlay of the docking pose of the capsaicin with the best energy cluster (orange stick) with the capsaicin with the best numerousness in the cluster (cyan stick) and the capsaicin bound to the TRPV1 agonist-bound conformation (pink stick); in cyan/beige and pink cartoon TRPV1 in antagonist- and agonist-bound conformation, respectively. Pointed by the red arrows the different orientations of the Arg 557 before and after the conformational change.

However, in the latter case, two possible capsaicin binding positions were detected: the one with the best energy is displaced with respect to the inhibitor position (Figure 3A) and shows an estimated nine-folds lower inhibition constant, and the other is characterised by a lower binding energy and is in the same conformation as the inhibitor and the capsaicin in active conformation (Figure 3A,B).

The side chain orientation of Arg557 on S4 plays a crucial role in receptor activation because H-bond formation with Glu 570 leads to the rotation of the S4–S5 linker where glutamine is located, performing one of the relevant conformation changes required for channel opening [43].

Capsaicin, unlike the inhibitor SB-366791, seems to play a role in the correct orientation of this residue by interacting directly with it [44]. The docking simulation highlighted how capsaicin can perform H-binding with Arg557 not only when TRPV1 is in an active conformation but also when it is in an inhibited one.

**Figure 4:**
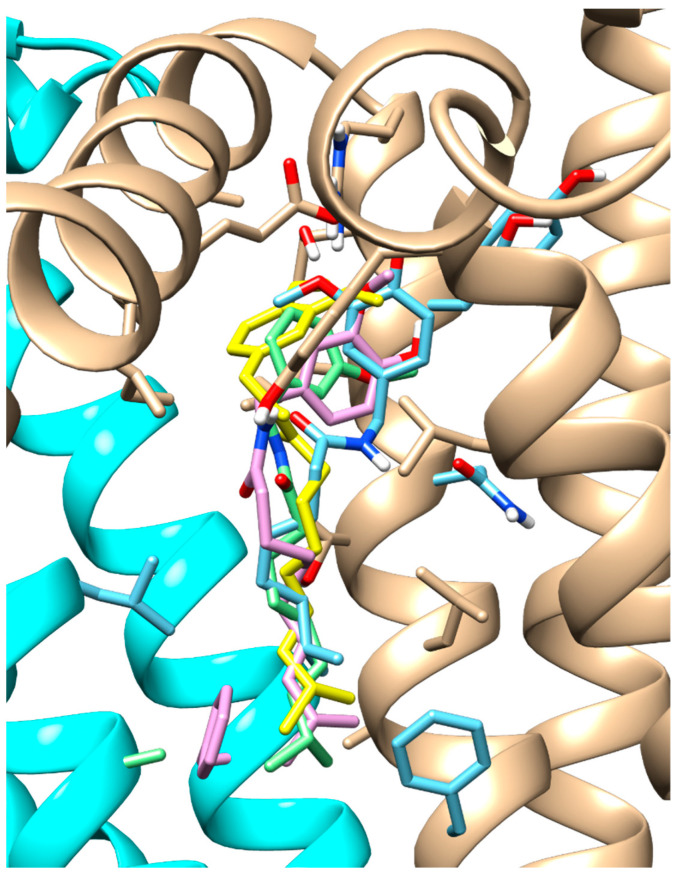
Docking results for capsaicinoids. Overlay of the docking pose of the capsaicin (green stick), dihydrocapsaicin (yellow stick), homocapsaicin (pink stick) and nordihydrocapsaicin (cyan stick). TRPV1, in agonist-bound conformation, is represented in cartoon, with cyan and beige colours for two different chains.

Among the remaining capsaicinoids tested, the only one with already known TRPV1 agonist activity is dihydrocapsaicin; for all the others, no pharmacological activity has been recorded [26]. The results showed that dihydrocapsaicin, homocapsaicin and nordihydrocapsaicin, in addition to sharing the same position in the pocket (Figure 4), also have comparable binding energies.

Notably, homocapsaicin seems to achieve even higher binding values than dihydrocapsaicin in both tested TRPV1 conformations: −7.96 Kcal/mol (model) and −7.14 Kcal/mol (antagonist-bound conformation) for homocapsaicin compared to −6.62 Kcal/mol (model) and −6.76 Kcal/mol (antagonist-bound conformation) for dihydrocapsaicin.

**Figure 5:**
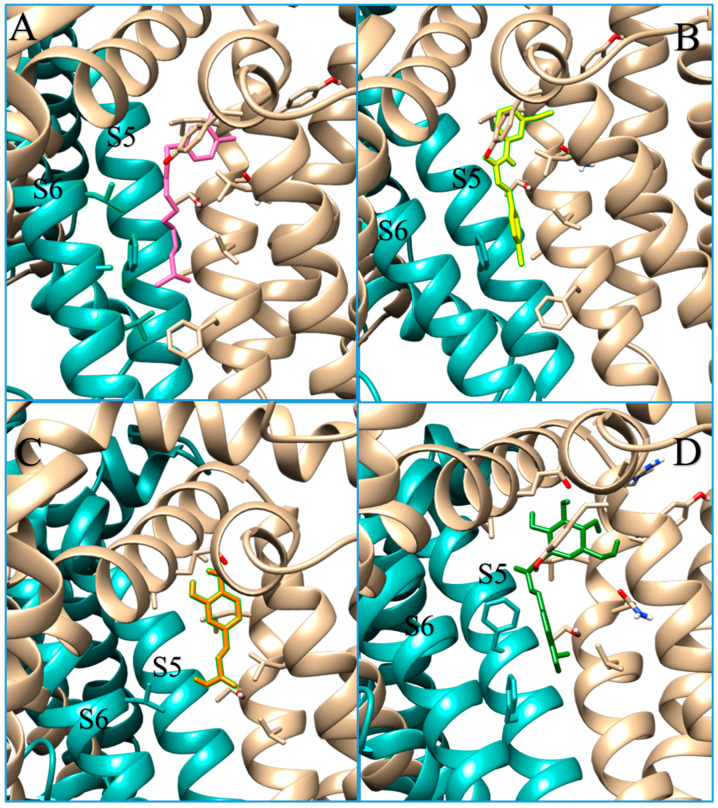
Docking results for TRPV1 agonist, antagonist, and caffeic acid hexoside. Docking interactions between TRPV1 in antagonist-bound conformation (cyan and beige depict chain A and B, respectively) and capsaicin (pink sticks in panel (**A**)), SB-366791 (yellow sticks in panel (**B**)), caffeic acid (orange sticks in panel (**C**)) and caffeic acid hexoside (green sticks in panel (**D**)). In beige and cyan, stick residues depict the interaction between the ligand s and the chain A and B, respectively. Caffeic acid hexoside, as well the caffeic acid, and the inhibitor SB-366791 do not interact with S6 helix.

Docking analysis for icariside E5 indicates that it does not appear to be a good ligand for TRPV1, due to its unfavourable binding energy to the receptor in the agonist-bound state and its higher estimated inhibition constant. The case of the caffeic acid hexoside is different, as the interaction energy for this compound is comparable to that of other capsaicinoids, especially for TRPV1 in the antagonist-bound state. Indeed, its role seems to be more comparable to that of an inhibitor. Caffeic acid is known for its inhibitory role in TRPV1 [45], which is why we decided to also perform docking simulations with this compound as a control. A common feature for inhibitors is the low number of interactions with the other chain at the interface and fewer interactions with the subunit S6, an active component of the pore domain (Figure 5). As can be seen from the interactions detected via the docking analysis, caffeic acid hexoside shows little interaction with the B or D chain. This may lead to a minor conformational change compared to that induced by capsaicin and may explain the minor/absent response of the receptor to this molecule, despite the appreciable position in the pocket, the energy of the interactions and the estimated inhibition constants.

The results obtained highlight that homocapsaicin, nordihydrocapsaicin and dihydrocapsaicin could have a similar effect as capsaicin on the TRPV1 receptor, representing a resource in the therapeutic field. Actually, the role of capsaicin via its modulation of TRPV1 is widely studied for its implications in the antidiabetes and antihypertension mechanisms [46]. Notably, the object of several studies is also the induction of TRPV1 via capsaicin which may result in glucose homeostasis regulation and a reduction in hyperinsulinemia with an enhancement of insulin sensitivity or may result in the regulation of cellular lipid content, inducing an increase in fatty acid oxidation through the TRPV1-induced calcium flux [47]. It is also noteworthy that the capsaicin application in analgesia exploits its ability after activation to desensitise the TRPV1 receptor present in the small fibre sensory afferent nerve endings [48].

## 3. Materials and Methods

### 3.1. Materials and Chemicals

Acetonitrile and methanol (all HPLC grade) were obtained from Merck (Darmstadt, Germany). Glacial acetic acid was purchased from Carlo Erba (Cornaredo, Milan, Italy). HPLC-grade water (18.2 MΩ) was prepared using a Millipore Milli-Q purification system (Millipore Corp., Bedford, MA, USA). Chlorogenic acid, quercitrin (quercetin-3-*O*-rhamnoside), isoquercitrin (quercetin-3-*O*-glucoside), rutin (quercetin-3-*O*-rutinoside), capsaicin and Folin-Ciocalteu reagent were purchased from Sigma-Aldrich (St. Louis, MO, USA).

### 3.2. Plant Materials and Sample Treatment

Three varieties of *Capsicum annuum* with different levels of pungency were analysed. These included the non-pungent variety Friariello (GP: green pepper), two different samples of the pungent Cayenne variety (RP1: red pepper 1; RP2: red pepper 2) and one pungent Dzuljunska Sipka variety (RP3: red pepper 3). GP and RP1 were purchased at the local markets of Avellino (AV), Italy (40°54.8964′ N and 14°47.4618′ E); RP2 was purchased at the local market of Grottaminarda (AV), Italy (41°4.1832′ N and 15°3.5364′ E); and RP3 was kindly provided by a farm located in Sicily (Southern Italy). Fresh pepper fruits were rapidly washed in distilled water, freeze-dried and kept at −20 °C until use. Once the peduncles were removed, the freeze-dried peppers were ground in a kitchen grinder and, for each variety, 0.5 g aliquots of ground sample were subjected to extraction with 5 mL of 80% aqueous methanol for 30 min in an ultrasonic bath (Astrason 10E, Farmingdale, NY, USA). After centrifugation (4000 rpm, 4 °C, for 10 min), the supernatant was removed, the pellet was suspended in 5 mL of 80% aqueous methanol and the extraction was repeated under the same conditions. The two supernatants were pooled and dried first under nitrogen flow and then in a rotary evaporator (LaboRota 4000/HB Efficient, Heidolph, Schwabach, Germany). Samples were stored at −20 °C until use.

### 3.3. Analysis of Total Phenolic Content

The amounts of total phenols in the pepper fruit extracts were determined according to the Folin–Ciocalteu method [49], using gallic acid as a reference standard. Folin–Ciocalteu’s reagent (62.5 μL) and 250 μL of distilled water were added to 62.5 μL of suitable aqueous dilutions of dry extracts. The reaction mixture was mixed and allowed to stand for 6 min. Finally, 625 μL of sodium carbonate 7.5% (*w*/*v*) and 500 μL of distilled water were added, and the solution was incubated in the dark for 90 min. The absorbances of the samples were measured at 760 nm. The total phenolic content was expressed as grams of gallic acid equivalents (GAE) per kilogram of dry weight (gGAE Kg^−1^ DW). All measurements were carried out in triplicate.

### 3.4. Analysis of Phenolic Compounds and Capsaicinoids Using Reversed-Phase High Performance Liquid Chromatography–Ultraviolet (RP-HPLC–UV) and HPLC-Electrospray Ionization Ion Trap Mass Spectrometry (HPLC-ESI-ITMS)

Dried samples were reconstituted in 0.1% acetic acid/methanol 80:20 and analysed using HPLC/ESI-ITMSn on a Surveyor MS micro-HPLC with a diode array detector and coupled with a LCQ DECA XP Max ion trap mass spectrometer, equipped with Xcalibur^®^ system manager data acquisition software (version 1.3; Thermo Finnigan, San Jose, CA, USA).

Phenolic compounds and capsaicinoids were separated on a Luna C18 (2) column (150 × 2 mm, 5 µm, 100 Å) manufactured by Phenomenex (Torrance, CA, USA), at a flow rate of 200 μL min^−1^; solvent A was 0.1% acetic acid in water, and solvent B was 0.1% acetic acid in acetonitrile. After holding for 2 min in 5% solvent B, elution was performed under the following conditions: 5 to 30% solvent B in 30 min, 30 to 45% solvent B in 13 min, 45 to 50% solvent B in 13 min and 50 to 95% solvent B in 5 min, followed by a 7 min hold.

The column effluent was split in two by a “T junction” placed after the chromatographic column and analysed “on-line” both using UV and ESI/MS; 80% of the effluent was sent to the UV detector (detection 280 nm) while 20% of the effluent was analysed via ESI/MS. Mass spectra were recorded from mass-to-charge ratio (*m*/*z*) 120 to 1600 in negative ionization mode. The capillary voltage was set at −10 V, the spray voltage at 3.0 kV, and the tube lens offset at −10 V. The capillary temperature was 275 °C. Data were acquired in MS, MS/MS and MSn scan modes.

### 3.5. Quantification of Capsaicinoids

The quantitative analyses of capsaicinoids present in the extracts of the three hot pepper samples (RP1, RP2 and RP3) were carried out through HPLC–UV, using an HP 1100 Series HPLC (Agilent, Palo Alto, CA, USA) equipped with a binary pump (G-1312A) and a UV detector (G-1314A). The chromatographic conditions were as described for the HPLC–MS system, except that phenols were separated on a XBridge BEH C18 column (130 Å, 5 mm, 4.6 mm × 150 mm) (Waters, Milford, MA, USA) at a flow rate of 1 mL min^−1^. The detection wavelength was 280 nm. The quantification of capsaicinoids was performed using external calibration curves generated via the repeated injections of a fixed volume of capsaicin standard solutions in a concentration range of 0.005–0.1 µg/µL with four different concentration levels and duplicate injections at each level. All samples were analysed in duplicate. The results were expressed as g kg^−1^ DW.

### 3.6. TRPV1 Model Construction

In order to obtain the 3D model of the human TRPV1 protein sequence (UniProt code: Q8NER1), homology modelling and fold recognition strategies were combined due to the complexity of the structure and the lack of templates for the whole sequence.

Multiple models were created by Modeller 9.22 [50] using three templates: one for the N-terminal region, one for the core and one for the C-terminal region of the protein sequence. In detail, the E3 ubiquitin–protein ligase (PDB code: 8D4X) was used as a template to model the first 126 residues. The template selected to model the core was the structure of TRPV1 from Rattus Norvegicus (PDB code: 7LPB) in the presence of capsaicin at 25 °C (85.83% identity). The C terminal region (residues 752 to 839) was modelled using the squirrel TRPV1 structure for residues from 753 to 779 (PDB code: 7LQY) as a template; the models of the regions in the ranges 780–812 and 813–839 were obtained by running Alpha Fold [51] and Phyre2 [52], respectively. Supplementary File S1 provides a detailed description of the modelling procedure and the alignments used as input files for Modeller.

The models were validated in terms of structural features by analysing the Z-scores computed by ProSA-web [53], the Q-means determined by QMEAN-SWISS-MODEL [54] and the Ramachandran plots generated by PROCHECK [55]. Finally, the model with the best features was selected. Supplementary File S2 is the final PDB file of the TRPV1 tetrameric structure.

### 3.7. Docking Simulations

The docking simulation procedure applied in this study is as follows. The PubChem database [56] was used to download in SDF format the structures of nordihydrocapsaicin (CID_168836), dihydrocapsaicin (CID_107982), homocapsaicin (CID_6442566), homodihydrocapsaicin (CID_3084336), icariside E5 (CID_91884923), caffeic acid (CID_689043) and caffeic acid hexoside (CID_6124135). The structure of capsaicin (CID_1548943) was also present in the PDB structure 7LPB). Structures in SDF format were converted to PDB format by Chimera (www.rbvi.ucsf.edu/chimera, accessed on 20 July 2023). For docking simulations, two target protein structures were used: the previously described model of human TRPV1 in an active conformation (Supplementary File S2) and the Cryo-EM structure of human TRPV1 in complex with the analgesic drug SB-366791 (PDB code: 8GFA) for the inactive form. The structures of ligands and proteins were prepared for docking by AutoDock Tool 1.5.6 [57]. The docking simulations were performed using a 64 × 68 × 80 grid and 0.375 Å spacing (gridcenter: 161.616–135.517–126.286 for the active conformation and 107.152–80.204–92.147 for the inactive one). Docking simulations were computed using AutoDock 4.2.5.1 [58] and ligand interactions were analysed using AutoDock Tool 1.5.6 and LigPlot+ v.2.2.8 [59].

## 4. Conclusions

In the three *Capsicum annuum* varieties Friariello, Cayenne and Dzuljunska Sipka, a total of 35 compounds belonging to different classes were identified and a quantitative analysis of capsaicinoids was performed. Furthermore, to the best of our knowledge, this is the first report describing the presence of apigenin-7-*O*-(malonylapiosyl) hexoside in the extracts of pungent pepper fruits. The Dzuljunska Sipka variety presented the highest values for phenolic and capsaicinoid content. The modelling, for the first time, of the complete 3D structure of the homotetrameric human TRPV1 offered the chance to evaluate the interaction of the detected compounds with a more comprehensive model. Results suggest that more compounds may interact as capsaicin at the binding site of the receptor and open the possibility for further studies on the possible activity of these compounds.

## Figures and Tables

**Table 1:** Total phenolic content in extracts from the four different *C. annuum* samples.

Sample	Total Phenols Content (g_GAE_/Kg DW)
GP	3.58 ± 0.02
RP1	5.24 ± 0.16
RP2	7.55 ± 0.05
RP3	8.91 ± 0.05

Abbreviations: GP (green pepper); RP1 (red pepper 1); RP2 (red pepper 2); RP3 (red pepper 3).

**Table 2:** List of compounds tentatively identified via HPLC-ITMS in the four different *C. annuum* samples, including quasi-molecular ions and fragment ions.

Peak	[M-H]^−^ *m*/*z*	MS/MS *m*/*z*	Identified Compound	GP	RP1	RP2	RP3
			***Phenolic compounds***				
**1**	341	179	Caffeic acid hexoside	x	x	x	x
**2**	337	191	Coumaroylquinic acid		x	x	x
**3**	325	163, 119	Coumaric acid hexoside	x	x	x	x
**4**	353	191, 179, 173	3-*O*-caffeoylquinic acid	x	x	x	x
**5**	355	337, 309, 265, 235, 217, 193, 175, 163	Ferulic acid hexoside	x	x	x	x
**6**	385	223, 205, 191	Sinapic acid hexoside	x	x	x	x
**7**	741	579, 285	Luteolin 7-(2″-pentosyl-4″-*O*-hexosyl)hexoside	x			x
**8**	355	337, 309, 265, 235, 217, 193, 175, 163	Ferulic acid hexoside		x	x	
**9**	579	561, 489, 459, 399, 369	Luteolin *C*-pentosyl-*C*-hexoside Isomer 1	x	x	x	x
**10**	593	575, 503, 473, 383, 353	Apigenin 6,8-di-*C*-glucoside (Vicenin-2)	x	x	x	x
**11**	579	561, 489, 459, 399, 369	Luteolin *C*-pentosyl-*C*-hexoside Isomer 2		x	x	
**12**	563	473, 443, 383, 353, 325, 297	Apigenin *C*-pentosyl-*C*-hexoside	x	x	x	x
**13**	563	473, 443, 383, 353, 325, 297	Apigenin 6-*C*-arabinoside-8-*C*-glucoside (Isoschaftoside)	x	x	x	x
**14**	563	473, 443, 383, 353, 325, 297	Apigenin 6-*C*-glucoside-8-*C*-arabinoside (Schaftoside)	x	x	x	x
**15**	521	359, 341, 329	Icariside E5	x	x	x	x
**16**	431	269, 225, 151, 149	Apigenin-*O*-hexoside				x
**17**	609	463, 447, 343, 301	Quercetin-*O*-rhamnosyl-*O*-hexoside	x		x	x
**18**	579	447, 285	Luteolin-*O*-(apiosyl)hexoside	x	x	x	x
**19**	463	301, 255, 179, 151	Quercetin-*O*-hexoside	x			x
**20**	593	285	Luteolin deoxyhexosylhexoside				x
**21**	563	269, 225	Apigenin 7-*O*-(2″-*O*-apiosyl)glucoside (Apiin)				x
**22**	447	429, 369, 357, 327, 299, 285, 255	Luteolin-*C*-hexoside				x
**23**	447	301, 179, 151	Quercetin-3-*O*-rhamnoside (Quercitrin)	x	x		x
**24**	433	301, 271, 151	Quercetin-*O*-pentoside				x
**25**	621 665	579, 561, 489, 447, 285 621, 579, 489, 285	Luteolin-*O*-(apiosyl-acetyl)hexoside Luteolin-*O*-(apiosyl-malonyl)hexoside	x	x	x	x
**26**	649	605, 563, 269	Apigenin 7-*O*-(malonylapiosyl) hexoside				x
			***Capsacinoids***				
**27**	292	277, 156	Nordihydrocapsaicin		x	x	x
**28**	304	289, 168	Capsaicin		x	x	x
**29**	306	291, 170	Dihydrocapsaicin		x	x	x
**30**	318	303, 182	Homocapsaicin		x	x	x
**31**	320	305, 184	Homodihydrocapsaicin		x	x	x
			***Other compounds***				
**32**	191	173, 171, 155, 127, 111, 109	Quinic acid	x	x	x	x
**33**	164	147	Phenylalanine	x	x	x	x
**34**	203	185, 159, 116	Tryptophan	x	x	x	x

Abbreviations: GP (green pepper); RP1 (red pepper 1); RP2 (red pepper 2); RP3 (red pepper 3). x = detected.

**Table 3:** Content of capsaicinoids in extracts from the three hot pepper samples (g/kg DW).

	RP1	RP2	RP3
Nordihydrocapsaicin	1.12 ± 0.02	0.28 ± 0.00	9.59 ± 0.12
Capsaicin	4.85 ± 0.09	2.62 ± 0.07	16.76 ± 0.14
Dihydrocapsaicin	4.12 ± 0.17	1.65 ± 0.07	12.36 ± 0.17
Homocapsaicin	0.15 ± 0.01	0.07 ± 0.00	1.00 ± 0.03
Homodihydrocapsaicin	0.26 ± 0.01	0.10 ± 0.00	1.04 ± 0.08
Total Capsaicinoids	10.51 ± 0.28	4.72 ± 0.14	40.75 ± 0.54

Abbreviations: RP1 (red pepper 1); RP2 (red pepper 2); RP3 (red pepper 3).

**Table 4:** Summary of the docking results. If the numerousness and best energy values are not attributable to a single cluster or if the cluster density values are very similar, both cluster IDs are reported. Residues involved in H-bonds are underlined and highlighted in bold.

Receptor	Ligand	Lowest Binding Energy (Kcal/mol)	Ki	No. in Cluster	Interacting Residues
Agonist-bound conformation	Capsaicin	−7.33	4.21 uM	62	Tyr511-Leu515-Phe543-Ala546-Leu547-Thr550-Asn551-Leu553-Tyr554-**Arg557**-Glu570-Ile573-Phe591(D)-Ala666(D)-Leu670(D)
	Dihydrocapsaicin	−6.76	11.14 uM	44	**Tyr511**-Leu515-Phe543-Ala546-Leu547-Thr550-**Leu553**-**Arg557**-Ala566-Ile569-Glu570-Ile573-Phe591(D)-Leu670(D)
	Nordihydro-capsaicin	−6.7	12.26 uM	7	Tyr511-Ser512-Leu515-Phe543-Ala546-Leu547-Thr550-**Asn551**-Tyr554-**Arg557**-Ala566-**Glu570**-Phe591(D)-Leu670(D)
		−6.36	21.86 uM	31	Tyr511-Leu515-Phe543-Ala546-Leu547-Thr550-**Leu553**-Tyr554-**Arg557**-Ala566-Ile569-Glu570-Ile573-Phe591(D)
	Homocapsaicin	−7.14	5.82 uM	15	Tyr511-Leu515-Phe543-Ala546-Leu547-Thr550-Tyr554-**Arg557**-**Ala566**-Il569-Glu570-Ile573-Phe591(D)-Leu663(D)-Ala666(D)-Leu670(D)
		−6.69	12.50 uM	12	**Tyr511**-Ser512-Leu515-Phe543-Ala546-Leu547-Thr550-Asn551-Tyr554-Arg557-Glu570-Ile573-Phe591(D)-Leu670(D)
	Homodihydro-capsaicin	−5.97	11.09 uM	30	**Tyr511**-Leu515-Phe543-Ala546-Leu547-Thr550-Asn551-Leu553-Tyr554-**Arg557**-Glu570-Ile573-Phe591(D)-Leu663(D)-Ala666(D)-Leu670(D)
	Caffeic acid hexoside	−6.31	23.55 uM	27	Tyr511-Leu515-Phe543-Ala546-Leu547-**Thr550**-**Asn551**-Leu553-**Tyr554**-**Arg557**-Ala566-Glu570-Phe591(D)
		−5.97	42.38 uM	29	Tyr511-Leu515-Phe543-Ala546-Leu547-**Thr550**-Asn551-Leu553-**Tyr554**-**Arg557**-Phe587(D)-Phe591(D)-Leu670(D)
	Icariside E5	−4.88	266.06 uM	2	Phe507-**Tyr511**-Leu515-Leu518-**Phe543**-Ala546-Leu547-Thr550-**Arg557**-Ala566-Ile569-**Glu570**-Ile573-Phe591(D)
	Caffeic acid	−5.09	186.59 uM	49	**Tyr511**-Leu515-Leu547-Thr550-Asn551-Leu553-Tyr554-**Arg557**
	SB-366791	−7.25	4.87 uM	97	Tyr511-Leu547-Thr550-**Arg557**-Ala566-Ile569-Glu570-Ile573-Phe591(D)-Leu670(D)
Antagonist-bound conformation	caffeic acid	−5.94	23.65 uM	75	Ser512-Leu515-Thr550-**Asn551**-Leu553-Tyr554-**Arg557**-Ala566-Val567-Glu570-**Gln701**
		−4.83	289.79 uM	1	Leu515-Leu547-**Thr550**-Leu553-**Ala566**-Ile569-Glu570-Leu670(B)
	capsaicin	−8.2	189.50 nM	25	Leu515-Phe543-Ala546-Leu547-Thr550-Leu553-Tyr554-Thr556-**Arg557**-Ala566-Val567-Glu570-Phe591-**Gln701**-Leu670(B)
		−7.77	704.99 nM	48	**Tyr511**-Leu515-Phe543-Ala546-Leu547-Thr550-Asn551-Leu553-**Tyr554**-Glu570-Leu663(B)-Ala666(B)-Phe591(B)-Leu670(B)
	caffeic acid hexoside	−6.43	1.79 uM	16	**Tyr511**-Leu515-Leu547-**Thr550**-**Asn551**-Leu553-Tyr554-**Arg557**-Ala566-Val567-Ile569-Glu570-Ile573-**Gln701**-Leu670(B)
		−5.61	21.30 uM	24	Tyr511-**Ser512**-Leu515-**Ala546**-Leu547-Thr550-**Asn551**-Leu553-**Tyr554**-**Ala566**-Ile569-**Glu570**-Phe587(B)-Phe591(B)
		−5.53	22.72 uM	32	**Tyr511**-Ser512-Leu515-Ala546-Leu547-**Thr550**-Asn551-Leu553-Tyr554-**Arg557**-**Ala566**-Phe587(B)-Phe591(B)-Leu670(B)
		−5.48	52.79 uM	6	Tyr511-Leu515-**Phe543**-Ala546-Leu547-**Thr550**-**Asn551**-Leu553-Tyr554-Ile569-Glu570-Ile573-Phe587(B)-Phe591(B)-Ala666(B)-Leu670(B)
	Homocapsaicin	−7.96	1.35 uM	29	**Tyr511**-**Ser512**-Leu515-Phe543-Ala546-Leu547-Thr550-**Asn551**-Leu553-**Tyr554**-Arg557-Ile569-Glu570-Phe587(B)-Phe591(B)-Leu663(B)-Ala666(B)-Leu670(B)
	Nordihydro-capsaicin	−6.59	4.91 uM	38	Tyr511-Ser512-Leu515-Phe543-Ala546-Leu547-**Thr550**-Asn551-Ala566-Ile569-**Glu570**-Phe591(B)-Leu663(B)-Leu670(B)
	Homodihydro-capsaicin	−6.58	4.44 uM	39	**Tyr511**-Ser512-Phe522-Phe543-Ala546-Leu547-Thr550-Asn551-Leu553-Tyr554-Ile569-**Glu570**-Ile573-Phe587(B)-Phe591(B)-Leu670(B)
	Dihydrocapsaicin	−6.62	2.53 uM	47	**Tyr511**-Ser512-Leu515-Phe543-Ala546-Leu547-Thr550-Asn551-Leu553-Tyr554-Ile569-**Glu570**-Ile573-Phe587(B)-Phe591(B)-Ala666(B)-Leu670(B)
	SB-366791	−8.12	923.53 nM	100	**Tyr511**-Ser512-Leu515-Phe516-Ala546-Leu547-Thr550-Asn551-Leu553-Tyr554-Ile573-Phe591(B)

## Data Availability

The data presented in this study are available on request from the corresponding authors.

## Supplementary Materials

The following supporting information can be downloaded at: https://www.mdpi.com/article/10.3390/molecules28196772/s1, Supplementary File S1: Detailed description of the procedure applied to generate a 3D model for the complete human TRPV1 sequence. Supplementary File S2: TRPV1_model as PDB file.

## Sample Availability

Samples of extracts used in the study are available from the authors.
