# Supplementary material for: Phenolic Compounds and Capsaicinoids in Three Capsicum annuum Varieties: From Analytical Characterization to In Silico Hypotheses on Biological Activity
Source: Molecules. 2023 Sep 22;28(19):6772. doi: 10.3390/molecules28196772 (PMC10574069; doi:10.3390/molecules28196772)
Supplement: Supplementary file 1 [file molecules-28-06772-s001.zip › supplementary_file-S1.pdf]

Phenolic compounds and capsaicinoids in three *Capsicum annuum* varieties: from analytical characterization to *in silico* hypotheses on biological activity

Deborah Giordano [1], Angelo Facchiano [1,*], Paola Minasi [1], Nunzio D'Agostino [2], Mario Parisi [3] and Virginia Carbone [1,*]

1 Institute of Food Sciences, National Research Council, via Roma 64, 83100 Avellino, Italy
2 Department of Agricultural Sciences, University of Naples Federico II, Portici, Naples, Italy
3 CREA Research Centre for Vegetable and Ornamental Crops, Via Cavalleggeri 25, 84098 Pontecagnano, Italy
* Correspondence: AF: angelo.facchiano@isa.cnr.it; VC: virginia.carbone@isa.cnr.it

Supplementary file S1

Detailed description of the procedure applied to generate a 3D model for the complete human TRPV1 sequence.

By submitting the N-terminal 126 residues of human TRPV1 sequence to Phyre2, it was obtained a model of the region from 18 to 90 based on the template PDB structure 8D4X. The model obtained is named here as "start". By making by Modeller9.22 a new model of the first 126 residues on the base of the alignment with region 1868-2301 of the PDB structure 8D4X, it was possible to obtain a different model, named here as "coil"(see fig.S1 for the .ali).

```
>P1;coil
sequence:coil:1          :A:126   :::::
MKKWSSTDLGAAADPLQKDTCPDPLDGDPNSRPPPAKPQLSTAKSRTRLFGKGDSEEAFPV-DCPHEEGELDS
CPTITVSPVITIQRPGDGPTGARLLSQDSVAASTEKTLRLYDRRSIFEAVAQN/
*

>P1;8d4x
structureX:8d4x:1868     :B:2301  :::::
----------------------------------PNHPLHASQNSARRMSARGDFAEAIPLADQPHDRNCLEV
LPT-KMSPPLTSFRPQCSFMGM-VISHDMLLGRWRLSLELFGRRSFYTAIAQ-/
*
```

Figure S1. The content of the .ali file used for the modelling of the "coil" model.

From a new file .ali exploiting as template the start and coil models, it was possible to obtain the model of the first 126 residues of the monomer of TRPV1 (see fig. S2 for the .ali), that is named here as “startM”.

```
>P1;startM
sequence:startM:1        :A:126   :::::
MKKWSSTDLGAAADPLQKDTCPDPLDGDPNSRPPPAKPQLSTAKSRTRLFGKGDSEEAFPVDCPHEEGELDSCPTITVSP
VITIQRPGDGPTGARLLSQDSVAASTEKTLRLYDRRSIFEAVAQN/
*

>P1;coil
structureX:coil:1        :A:126   :::::
MKKWSSTDLGAAADPLQKDTCPDPLDGDPNSRPPPAKPQLSTAKSRTRLFGKGDSEEAFPVDCPHEEGELDSCPTITVSP
VITIQRPGDGPTGARLLSQDSVAASTEKTLRLYDRRSIFEAVAQN/
*

>P1;start
structureX:start:18      ::90     :::::
-----------------KDTCPDPLDGDPNSRPPPAKPQLSTAKSRTRLFGKGDSEEAFPVDCPHEEGELDSCPTITVSP
VITIQRPGDG-----------------------------------/
*
```

Figure S2. The content of the .ali file used for the modelling of the “startM” model.

To model the C-terminal region (752-839) four different templates have been used. The PDB structure 7LPB, from 719 to 752, to prepare a connection with the core region, the PDB structure 7LQY, which cover the sequence only until residue 779; the model named “alpha”, i.e. the AlphaFold predicted structure for region 780-812, and the model named “tail”, that is the Phyre2 prediction for the region from 813 to 839 . The alignment used for the construction of the tetrameric model of the C-terminal region is shown in fig. S3. The model obtained was named as “Cter”.

```
> P1;Cter
AFRSGKLLQVGYTPDGKDDYRWCFRVDEVNWTTWNTNVGIINEDPGNCEGVKRTLSFSLRSSRVSGRHWKNFALVPLLRE
ASARDRQSAQPEEVYLRQFSGSLKPEDAEVFKSPAASGEK

>P1;7lpb
AFRSGKLLQVGFTPDGKDDYRWCFRVDEVNWTTW----------------------------------------------
----------------------------------------

>P1;7lqy
AFRSGKLLQVGYTPDGKDDYRWCFRVDEVNWTTWNTNVGIINEDPGNCEGVKRTLSFSL---------------------
----------------------------------------

>P1;alpha
-----------------------------------------------------------RSSRVSGRHWKNFALVPLLRE
ASARDRQSAQPEE---------------------------

>P1; tail
--------------------------------------------------------------------------------
-------SAQPEEVYLRQFSGSLKPEDAEVFKSPAASG—
```

Figure S3. The alignment used for the setup of the .ali file used for the modelling of the “Cter” model.

Finally, to obtain the complete model of the human TRPV1 receptor, another modelling step was performed exploiting the monomeric model obtained for the N-terminal region (startM), the template 7LPB for the core region and the model "Cter" for the last part of the sequence. The sequence alignment exploited for the creation of the file .ali for modeling the homotetramer is shown in fig. S4. It is important to underline that for achieving the best quality model, modelling procedure was not limited to the first model obtained as result. From this alignment, in fact, ten different models have been constructed, from which the best one was chosen for further refinement performing a remodeling procedure. The latter has exploited the alignment with the best model obtained, and with "7LPB"sequence. This results in further ten models, from which it was chosen the best one, that has been energetically minimized.

The final model of the human TRPV1 receptor is available as Supplementary file S2.

```
>P1;TRPV1
MKKWSSTDLGAAADPLQKDTCPDPLDGDPNSRPPPAKPQLSTAKSRTRLFGKGDSEEAFPVDCPHEEGELDSCPTITVSP
VITIQRPGDGPTGARLLSQDSVAASTEKTLRLYDRRSIFEAVAQNNCQDLESLLLFLQKSKKHLTDNEFKDPETGKTCLL
KAMLNLHDGQNTTIPLLLEIARQTDSLKELVNASYTDSYYKGQTALHIAIERRNMALVTLLVENGADVQAAAHGDFFKKT
KGRPGFYFGELPLSLAACTNQLGIVKFLLQNSWQTADISARDSVGNTVLHALVEVADNTADNTKFVTSMYNEILMLGAKL
HPTLKLEELTNKKGMTPLALAAGTGKIGVLAYILQREIQEPECRHLSRKFTEWAYGPVHSSLYDLSCIDTCEKNSVLEVI
AYSSSETPNRHDMLLVEPLNRLLQDKWDRFVKRIFYFNFLVYCLYMIIFTMAAYYRPVDGLPPFKMEKT-GDYFRVTGEI
LSVLGGVYFFFRGIQYFLQRRPSMKTLFVDSYSEMLFFLQSLFMLATVVLYFSHLKEYVASMVFSLALGWTNMLYYTRGF
QQMGIYAVMIEKMILRDLCRFMFVYIVFLFGFSTAVVTLIEDGKNDSLPSESTSHRWGPACRPPDSSYNSLYSTCLELF
KFTIGMGDLEFTENYDFKAVFIILLLAYVILTYILLLNMLIALMGETVNKIAQESKNIWKLQRAITILDTEKSFLKCMRK
AFRSGKLLQVGYTPDGKDDYRWCFRVDEVNWTTWNTNVGIINEDPGNCEGVKRTLSFSLRSSRVSGRHWKNFALVPLLRE
ASARDRQSAQPEEVYLRQFSGSLKPEDAEVFKSPAASGEK

>P1;startM
structureX:7start1:128     :A:252   :::::
MKKWSSTDLGAAADPLQKDTCPDPLDGDPNSRPPPAKPQLSTAKSRTRLFGKGDSEEAFPVDCPHEEGELDSCPTITVSP
VITIQRPGDGPTGARLLSQDSVAASTEKTLRLYDRRSIFEAVAQN-----------------------------------
--------------------------------------------------------------------------------
--------------------------------------------------------------------------------
--------------------------------------------------------------------------------
--------------------------------------------------------------------------------
--------------------------------------------------------------------------------
--------------------------------------------------------------------------------
--------------------------------------------------------------------------------
--------------------------------------------------------------------------------
----------------------------------------

>P1;7lpb
--------------------------------------------------------------------------------
---------------------------------DRRSIFDAVAQSNCQELESLLPFLQRS------------ETGKTCLL
KAMLNLHNGQNDTIALLLDVARKT---KQFVNASYTDSYYKGQTALHIAIERRNMTLVTLLVEN---VQAAANGDFFK--
--RPGFYFGELPLSLAACTNQLAIVKFLLQNSWQPADISARDSVGNTVLHALVEVADNTVDNTKFVTSMYNEILILGAKL
HPTLKLEEITNRKGLTPLALAASSGKIGVLAYILQREIHEPECRHLSRKFTEWAYGPVHSSLYDLSCIDTCEKNSVLEVI
AYSSSETPNRHDMLLVEPLNRLLQDKWDRFVKRIFYFNFFVYCLYMIIFTAAAYYRPVEGLPPYKLKNTVGDYFRVTGEI
LSVSGGVYFFFRGIQYFLQRRPSLKSLFVDSYSEILFFVQSLFMLVSVVLYFSQRKEYVASMVFSLAMGWTNMLYYTRGF
QQMGIYAVMIEKMILRDLCRFMFVYLVFLFGFSTAVVTLIEDG-----------------------NSYNSLYSTCLELF
KFTIGMGDLEFTENYDFKAVFIILLLAYVILTYILLLNMLIALMGETVNKIAQESKNIWKLQRAITILDTEKSFLKCMRK
AFRSGKLLQVGFTPDGKDDYRWCFRVDEVNWTTW----------------------------------------------
----------------------------------------

>P1;Cter
--------------------------------------------------------------------------------
--------------------------------------------------------------------------------
--------------------------------------------------------------------------------
--------------------------------------------------------------------------------
--------------------------------------------------------------------------------
--------------------------------------------------------------------------------
--------------------------------------------------------------------------------
--------------------------------------------------------------------------------
--------------------------------------------------------------------------------
AFRSGKLLQVGYTPDGKDDYRWCFRVDEVNWTTWNTNVGIINEDPGNCEGVKRTLSFSLRSSRVSGRHWKNFALVPLLRE
ASARDRQSAQPEEVYLRQFSGSLKPEDAEVFKSPAASGEK
```

Figure S4. The alignment used for the setup of the .ali file used for the modelling of the "complete TRPV1 model.
